# Increase of *O*-Glycosylated Oncofetal Fibronectin in High Glucose-Induced Epithelial-Mesenchymal Transition of Cultured Human Epithelial Cells

**DOI:** 10.1371/journal.pone.0060471

**Published:** 2013-04-12

**Authors:** Frederico Alisson-Silva, Leonardo Freire-de-Lima, Joana L. Donadio, Miguel C. Lucena, Luciana Penha, Julliana N. Sá-Diniz, Wagner B. Dias, Adriane R. Todeschini

**Affiliations:** Instituto de Biofísica Carlos Chagas Filho, Universidade Federal do Rio de Janeiro, Rio de Janeiro, Brasil; Faculdade de Medicina, Universidade de São Paulo, Brazil

## Abstract

Growing evidences indicate that aberrant glycosylation can modulate tumor cell invasion and metastasis. The process termed "epithelial-mesenchymal transition" (EMT) provides a basic experimental model to shed light on this complex process. The EMT involves a striking decline in epithelial markers, accompanied by enhanced expression of mesenchymal markers, culminating in cell morphology change and increased cell motility. Few recent studies have established the participation glycosylation during EMT. Studies now come into knowledge brought to light the involvement of a site-specific O-glycosylation in the IIICS domain of human oncofetal fibronectin (onfFN) during the EMT process. Herein we show that high glucose induces EMT in A549 cells as demonstrated by TGF-β secretion, cell morphology changes, increased cellular motility and the emergence of mesenchymal markers. The hyperglycemic conditions increased onfFN protein levels, promoted an up regulation of mRNA levels for ppGalNAc-T6 and FN IIICS domain, which contain the hexapeptide (VTHPGY) required for onfFN biosynthesis. Glucose effect involves hexosamine (HBP) biosynthetic pathway as overexpression of glutamine: fructose-6-phosphate amidotransferase increases mesenchymal markers, onfFN levels and mRNA levels for FN IIICS domain. In summary, our results demonstrate, for the first time that the metabolism of glucose through HBP promotes O-glycosylation of the oncofetal form of FN during EMT modulating tumorogenesis.

## Introduction

Glucose, as a major energy source, provides ATP and various macromolecules required for cancer cell growth. In addition, glucose metabolism selectively affects genes expression [1]. Cancer cells exhibit a high rate of aerobic glycolysis even under normal oxygen concentration [2]–[4]. This metabolic shift involves increased glucose uptake to meet energy needs, and, it is a critical aspect supporting cancer phenotypes. Changes in glucose metabolism and uptake also alter distinct nutrient signaling pathways, including mammalian target of rapamicin (mTOR), AMP-activated protein kinase and hexosamine biosynthetic pathway (HBP) [1]. Indead, 2–5% of glucose entering cells is shunted through the HBP via conversion of fructose-6-phosphate to glucosamine-6-phosphate by the rate-limiting enzyme glutamine:fructose-6-phosphate amidotransferase (GFAT) [5]. Although flux through the HBP is likely increased in cancer cells as result of upregulated glucose uptake, the role for HBP in oncogenesis has been poorly explored. Importance of HBP is incontestable as its end-product UDP-GlcNAc and its derivates, UDP-GalNAc, UDP-ManNAc, and CMP-Neu5Ac (products of the action of epimerases and other enzymes) are crucial for *N*- and *O*-glycosylation of proteins [6] and alteration of the pool of activated substrates might lead to different glycosylation [7].

Changes in the glycosylation status of cell are common features of malignant transformation and tumor progression. Alteration of metabolic regulation of glycoconjugate biosynthesis [8]–[10] is result of initial oncogenic transformation, as well as a key event in induction of invasion and metastasis. Recent studies on epithelial-mesenchymal transition (EMT) have aided to shed light in the elucidation of the mechanisms involved in modulation of tumor cell invasion and metastasis [11]. The participation of glycolipids [12], [13] glycosyltranferases [14], [15] and intracellular O-GlcNAc [16] during EMT were recently demonstrated.

EMT is widely recognized in cancer progression by allowing a polarized epithelial cell to assume a mesenchymal cell phenotype, which includes enhanced migratory capacity, invasiveness, elevated resistance to apoptosis, and greatly increased production of extracellular matrix components (ECM) [11],[10]. Key targets of the pathways that induce EMT include a striking decline in epithelial markers, such as E-cadherin, desmoplakin, and cytokeratins, accompanied by enhanced expression of mesenchymal markers, such as vimentin, N-cadherin (N-cad) and fibronectin (FN) culminating in cell morphology change and increased cell motility [11],[17].

The FN has been broadly used as one of the mesenchymal markers, whose expression is strongly enhanced during EMT process [11],[17]. FN is a high-molecular-weight extracellular matrix glycoprotein that binds to membrane-spanning receptor proteins and therefore plays a major role in cell adhesion, growth, migration and differentiation[18]. FN exists in multiple isoforms that are formed through alternative splicing of the pre-mRNA from a single gene [19]. Twenty isoforms of human FN can be generated as a result of this cell type-specific splicing of the primary transcript. The mature FN molecules comprise a series of repeating amino acid sequences known as FI, FII and FIII structural domains [19]. Between FI and FIII domains there is a variable region (V or IIICS domain), which can generate 5 different variants after the alternative splicing (V0, V64, V89, V95, and V120) [20]. All variants, except V0 may contain the hexapeptide (VTHPGY) which can be glycosylated on its Thr residue by an UDP-GalNAc:polypeptide N-acetylgalactosaminyltransferase (ppGalNAc-T), creating the oncofetal epitope required for mAb FDC-6 binding [21], [22]. FDC6-positive FN was therefore termed "oncofetal fibronectin" (onfFN) [23]. The rate limiting step for the formation of onfFN is the addition of α-GalNAc to the Thr of the hexapeptide sequence VTHPGY by a specific ppGalNAc-T [23]. Recent work has demonstrated that up regulation of the expression of the ppGalNAc-T6 enhances transformational potentials of mammary epithelial cells through *O*-glycosylation of FN that may facilitate disruptive and invasive cell proliferation *in vivo* [14]. Freire-de-Lima and coworkers demonstrated that onfFN was up-regulated in human prostate epithelial cells undergoing EMT after TGF-β treatment. In this work the authors showed that EMT is totally dependent of onfFN appearance, once the knockdown of ppGalNAc-T3 and -T6, enzymes involved in the synthesis of onfFN was able to abrogate the EMT induction [22].

Taken together, these findings motivate us to investigate the role of high glucose concentrations in the regulation of the onfFN biosynthesis during EMT process. Herein, we demonstrate that high glucose concentration induces EMT and increases *O*-glycosylation of FN, which generates the onfFN, through HBP, modulating the tumorogenesis.

## Materials and Methods

### Cells culture

A549 cells, were purchased from American Type Culture Collection (ATCC, USA). Cells were seeded in 6-well plates (2.0×10^5^) and cultured in glucose-free Dulbecco's modified Eagle's medium (DMEM) (Gibco, UK) supplemented with 5 mM D(-)glucose (Sigma Chemical CO, USA), 10% fetal bovine serum (FBS), 100 IU/mL penicillin and 100 µg/mL streptomycin. After 24 h, the medium was harvested and fresh medium with 5 mM glucose (Normoglycemic; NG), 25 mM glucose (Hyperglycemic; HG) or 5 mM glucose +20 mM Mannitol (Osmotic Control or Osmoglycemic; OG) was added. Additionally, the cells were treated with or without 2 ng/mL TGF-β and incubated at 37°C in 5% CO_2_ for 48 h. FCCR-1-2813/FDC-6 hybridoma cells (FDC-6), which produces mAb directed against onfFN was purchased from ATCC, and maintained in RPMI 1640 medium (Gibco, UK) with 10% FBS. A549 cells were transiently transfected with GFAT plasmid (Origene Technologies, USA) using lipofectamine 2000 (Invitrogen, USA) as described [24]. In order to investigate the role of transforming growth factor beta (TGF-β) in onfFN biosynthesis, we incubated the A549 cells with 10 ng/mL of rabbit anti-TGF-β antibody (Santa Cruz Biotechnology, USA).

### Elisa for TGF-β measurement

Fresh culture supernatants from A549 cells maintained in NG, HG or OG conditions were recovered and assayed immediately with a human TGF-β duo set kit (R&D Systems, USA). DMEM containing 10% FBS was used as an internal control to normalize TGF-β amounts.

### Immunoprecipitation of onfFN and de-*O*-glycosylation

Five bottles of 75 cm^2^ of A549 cells growing in hyperglycemia were lysate with 10 mL of lysis buffer (50 mM de Tris-HCl pH 7.4; 0,5% NP-40; 250 mM NaCl; 5 mM EDTA e 50 mM de NaF) containing freshly added protease inhibitor solution (SIGMA). The lysate was incubated with anti-onfFN (FDC-6) for 90 min at room temperature followed by incubation with 60 µL of agarose-conjugated G Protein (SIGMA) for 120 min at room temperature. The lysates were washed, boiled at 100°C during 5 min and centrifuged at 14.000 rpm for 5 min to recover the supernatants of immunoprecipitation. The resulting material were submitted to non-denaturating de-*O*-glycosylation reaction using the glycoprotein deglycosylation kit (Calbiochem) as manufacturer instructions. Briefly, 1 µL of each glycosidase α2-3,6,8,9-neuraminidase, β1,4-galactosidase, endo-α-*N*-acetylgalactosaminidase and β-*N*-acetylglucosaminidase were added to the immunoprecipitated material and incubation proceed at 37°C for 26 h. After incubation, 10 µL of each reaction were used to western blot analysis.

### Immunoblotting

Samples were separated on 10% SDS-polyacrylamide gels, and were subsequently electro blotted to nitrocellulose membranes. The membranes were blocked in Tris-buffered saline with 0.1% (v/v) Tween 20 containing 3% (w/v) nonfat dry milk. The blocked membranes were then incubated overnight at 4 °C with primary antibodies against N-cad (IgG1, Santa Cruz, USA), vimentin (IgM; Sigma, USA), GFAT (Cell Signaling Technology, USA), Glyceraldehyde 3-phosphate dehydrogenase, GAPDH (Santa Cruz, USA), total FN (EP5, IgG1; Santa Cruz, USA) and FDC6, directed to onfFN [23]. FDC6 does not react with FN from plasma or from adult normal tissues [23],[25]. The blots were then washed, incubated with the appropriate secondary antibody, and developed using ECL (GE Healthcare, USA). ImageJ software was used for densitometry analysis of immunoblots, and all measurements were normalized against GAPDH loading controls.

### Cell circularity

Circularity ratio (C) was calculated as **C = P/(4πA)^0.5^**. Where P and A are, respectively, the perimeter and area of the cell [26].

### Cell motility analysis

Cell motility was determined as the area of phagokinetic tracks on gold sol particle-coated plates as described [27]. Briefly, A549 cells were seeded in 6-well plate, treated as described above, and maintained at 37°C in 5% CO_2_ for 48 h. After incubation, the cells were harvested with trypsin/EDTA, and 5×10^2^ cells in 1.0 mL of culture medium were seeded onto gold sol-coated wells (24-well plates). After 18 h cells were observed, and photographed using a light microscope (Olympus, USA). Motility track area of 15 cells/well were measured by Scion image program and expressed as square pixels [28].

### Determination of mRNA levels by real-time quantitative PCR (qRT-PCR)

The relative copy number from selected transcripts of three independent biological experiments was determined by qRT-PCR analysis to confirm differences of genes expression. Total RNA of 90% confluent cultured cells were extracted and purified using Qiagen RNeasy Mini Kit (Qiagen, USA). The cDNA were prepared from 2 µg of total RNA using RevertAid First Strand cDNA Synthesis Kit (Thermo Scientific, USA) with oligo-dT primer, according to manufacturer's instructions. Real-time quantitative PCR (qRT-PCR) were performed using SYBRGreen QRT-PCR Kit plus ROX (Thermo ABgene) according manufacturer protocol. Following primer pairs were used: GalNAc T-6: (sense, 5′-GCGTGATCATTGTGTTCCAC-3′; antisense, 5′-CGTACTGCTCCAGCTTCTCC-3′); IIICS domain of FN [29] (sense, 5′-GAATAATCAGAAGAGCGAGCC-3′; antisense, 5′-ACTCAGAAGTGTCCTGGAATG-3′); and β-actin (sense, 5′-CCACTCCCAGGGAGACCAAA-3′; antisense, 5′-TGAAGGTGACAGCAGTCGGTTGG-3′). Each pair of primer was designed from two exons separated by an intron. Amplification was carried out according to the following protocol: initial enzyme activation 95°C for 20 s, followed by 40 cycles 95°C for 3 s and 60°C for 30 s. The amount of fluorescence was detected using a LINEGENE 9600 (BIOER, Japan) machine. The number of PCR cycles (cycle threshold-Ct) required to reach fluorescence intensity above threshold was calculated using the software of LINEGENE 9600 (BIOER, Japan). The measurement of the unrelated control mRNA β-actin, was used to normalize the samples. The mean Ct value for three replicates of each gene was subtracted from the mean Ct value for three replicates of the reference β-actin gene in each sample to obtain ΔCt. The relative expression values (2^−ΔΔCt^) were used only for graphic construction. All statistical analyses were performed using Microsoft Office Excel program (Microsoft, USA).

## Results

### High glucose induces A549 cells to undergo EMT

**Figure 1:**
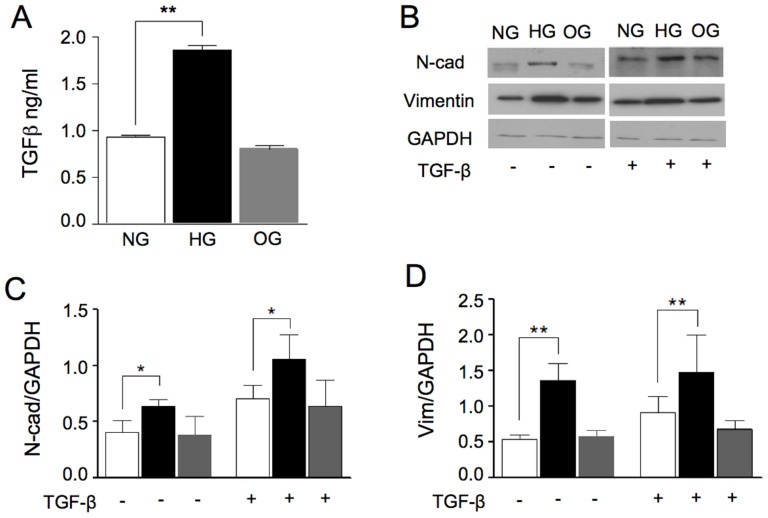
TGF-β production and expression of mesenchymal markers in A549 cells. (**A**) Quantification of TGF-β in supernatants from cells cultured for 48 h in NG (white bar), HG (black bar) or OG (gray bar). (**B**) Western blot of cell lysates loads analyzing expression levels of N-cad, (first lane) and Vimentin (second lane) in cells cultured in NG (white bar), HG (black bar) or OG (gray bar) conditions with or without 2 ng/mL TGF-β. Signal intensities were normalized, with GAPDH as loading control, and relative intensities of N-cad (**C**) and Vimentin (**D**) are shown. The results are representative of 3 independent experiments. Quantitative analyses are shown as mean ± standard deviation. P values were calculated using the Student's t test. *P≤0.01; **P*≤0.005*.

To access whether high glucose could induce the secretion of TGF-β in A549 cells, the cells were incubated in NG, HG, or OG conditions. In agreement with previous results [30], high glucose concentrations induced an increase in TGF-β secretion (Fig. 1A). The exposure of A549 cells to HG for 48 h increases the TGF-β levels when compared with NG or OG conditions. Since TGF-β is an important inductor of EMT, the increased secretion of TGF-β observed in HG condition (Fig. 1A) motivated us to investigate the expression of the mesenchymal phenotypic markers (Fig. 1B). Both N-cad (Fig. 1C) and vimentin (Fig. 1D) levels were significantly enhanced in cells exposed to HG condition and cells treated with TGF-β, while no differences in these markers were observed in cells under NG or OG conditions (Fig. 1B).

**Figure 2:**
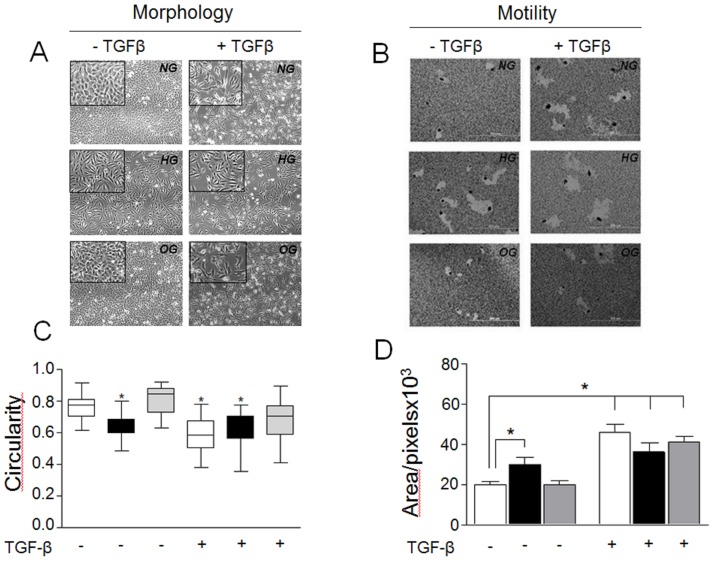
Analysis of cell morphology and motility. Cell morphology (**A**); cell motility (**B**) and cell circularity (**C**) of A549 cells treated in NG, HG or OG conditions with (right panel) or without TGF-β (left panel). Representative photos are presented. Tracks of 50 random individual cells on gold solution (**D**) were measured using the Scion Image program represented as squared pixels, and are shown as mean ± SD. NG (white bar), HG (black bar) or OG (gray bar). *P≤0,005.

In addition, our results show that exposure of A549 cells to HG for 48 h resulted in phenotypic conversion from epithelial cells into fibroblast-like cells (Fig. 2A, left panels), as observed for TGF-β-treated cells (Fig. 2A, right panels). Conversely, cells incubated in NG or OG exhibited a typical epithelial shape. Changes in cell morphology correlated with changes in cell circularity as observed in Fig. 2C. Cells under HG conditions or TGF-β treatment presented as thin fibroblast-like cells with circularity ratios significantly diminished when compared with the shape of epithelial cells in NG and OG medium, which have circularity ratios approaching one, resembling a circle (Fig. 2C).

Furthermore, we observed that cell motility, as determined by haptotaxis on gold sol-coated plates, was significantly enhanced by HG condition or by TGF-β treatment (Fig. 2B,D). In contrast, no clear change on cell motility was observed when cells were exposed to NG or OG conditions, and no synergistic effect with TGF-β was observed with the HG conditions. These results strongly suggest that HG environment activates the EMT process in A549 cells.

### Effect of HG on onfFN synthesis

**Figure 3:**
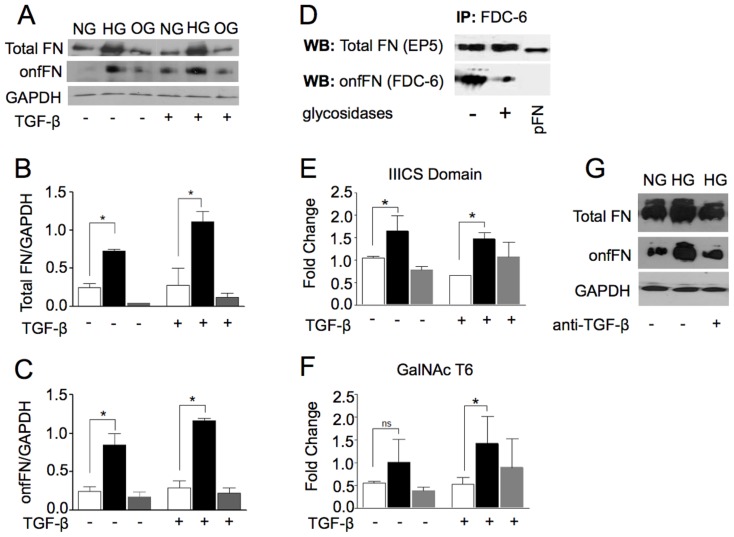
Effect of hyperglycemia onfFN biosynthesis. Western blot of A549 cell lysates cultured for 48 h in NG (white bar), HG (black bar) or OG (gray bar) medium with (+) or without (−) TGF-β, showing expression of total FN (first lane) and onfFN (second lane) (**A**). Signal intensities were normalized, with GAPDH as loading control, and relative intensities of total FN (**B**) and onfFN (**C**) are shown. (**D**) Western blot of A549 total FN (first lane) and onfFN (second lane) immunoprecipitated from cell lysates by FDC-6 mAbs, submitted (+) or not (−) to the remotion of *O*-glycosylation. Human plasma FN (pFN, 0.5 µg) was used as control. qRT-PCR analysis of gene that codifies IIICS domain of onfFN (**E**) and GalNacT6 (**F**) respectively. Graph shows one of three independent experiments as mean ± SD. * *P≤0.005*. Effect of anti-TGF-β blocking antibody in the expression of total FN (first line) and onfFN (second line) (**G**).

It has been well-accepted that FN is up-regulated in the EMT process [11],[17]. The onfFN is defined by the addition of the GalNAc to the Thr of the IIICS domain of FN, the rate limiting step for the recognition of onfFN by FDC-6, that only recognize the glycosylated isoform of FN [23] (Fig. 3). Recently, Freire-de-Lima and coworkers [22] demonstrated the up-regulation of onfFN during the EMT. Here, we demonstrate that high glucose concentration up-regulated onfFN levels (Fig. 3A,C) and consequently, total FN (Fig. 3A,B). FDC-6 mAb reacts selectively with FN that carries *O*-glycans since treatment of immunoprecipitated FN with exoglycosidases and with endo-α-N-acetylgalactosaminidose significantly decreases FDC-6 mAb activity when compared with non-deglycosylated control (Fig. 3D). As expected, de-*O*-glycosylation of immunoprecipitated FN did not influence the recognition by EP-5 antibody (Fig. 3D). Whereas human plasma FN (pFN), used as control, was detected by mAb EP5, but not by mAb FDC6 (Fig. 3D right panel). These results confirm the specificity of FDC-6 antibody to the glycosylated isoform of FN. Noteworthy was that HG treatment up-regulated the levels of FN mRNA splice forms containing the IIICS domain (onfFN) (Fig. 3E). HG treatment up-regulated the mRNA levels of ppGalNac-T6 (Fig. 3F), one of the enzymes involved in the biosynthesis of onfFN, and this effect was enhanced with the exogenous addition of TGF-β (Fig. 3C). The effects of HG were not due to osmolar changes, as 20 mM of manitol plus 5 mM of glucose (OG) had no significant effect on mRNA levels of the IIICS domain-containing FN splice forms or ppGalNAc-T6. Once it has been shown that TGF-β is involved in the up-regulation of IIICS domain of FN and ppGalNac-T6 in human epithelial cells [22] we further evaluated if the expression of TGF-β is required for the high glucose-induced onfFN biosynthesis. Fig. 3G shows that neutralization of TGF-β partially rescues the HG-induced onfFN expression, which indicates that the activation of onfFN biosynthesis by hyperglycemia involves, in part, TGF-β activation (Fig. 3G).

### HG induced onfFN synthesis and EMT is mediated by the HBP

**Figure 4:**
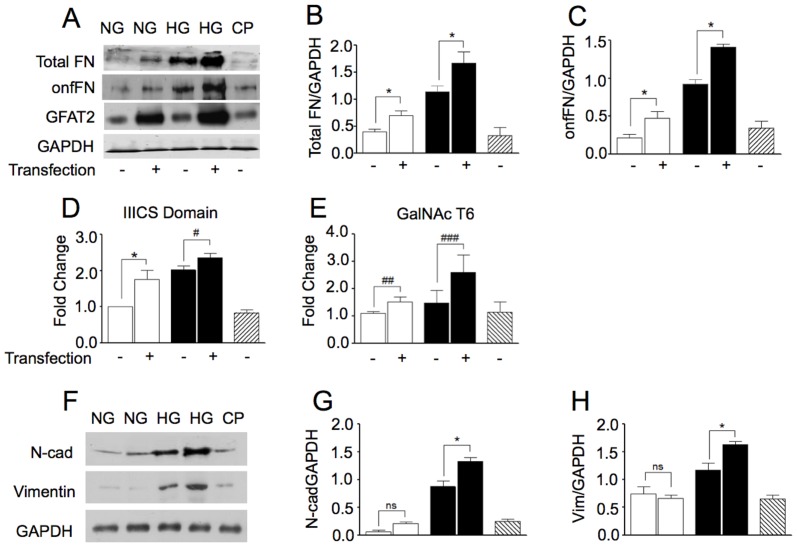
Effect of GFAT2 over expression in onfFN biosynthesis and expression of mesenchymal markers. A549 cells were transfected with expression vectors encoding GFAT2 (+) or empty expression vector (−), and cultured in NG (white bars), HG (Black bars) or NG plus Lipofectamine 2000 (CP, Scratched bars). Western blot analysis of expression of total FN (first lane), onfFN (second lane), GFAT (third lane) and GAPDH (**A**). The results are representative of 3 independent experiments. Signal intensities were normalized, with GAPDH as loading control, and relative intensities of total FN (**B**) and onfFN (**C**) are shown. qRT-PCR analysis of gene that codifies IIICS domain of onfFN (**D**) and GalNNcT6 (**E**). Western blot of cell lysates expression levels of N-cad, (first lane) and Vimentin (second lane) in cells cultured in NG (white bar), HG (black bar) or OG (gray bar) conditions with or without transfection of GFAT2 (+), or empty expression vector (−), or NG plus Lipofectamine 2000 (CP, Scratched bars) (**F**). Signal intensities were normalized, with GAPDH as loading control, and relative intensities of N-cad (**G**) and Vimentin (**H**) are shown. Quantitative analyses are shown as mean ± standard deviation. P values were calculated using the Student's t test. * P≤0.05; # P = 0.18; ## P = 0.14; ### P = 0.

Given that HBP provides the substrate UDP-GalNAc for the biosynthesis of onfFN, we further explored if the flux of glucose metabolism through HBP is involved in the increase of onfFN. To determine whether cellular onfFN expression is dependent on HBP, A549 cells were transfected with GFAT expression vector, leading to high expression of GFAT protein as compared with control cells (Fig. 4A). Immunoblotting of A549 cells cultured for 48 h in NG or HG conditions showed that the amount of onfFN in (Fig. 4C) and concequently total FN (Fig. 4B) in cellular extracts was increased by over-expression of GFAT, which indicates the involvement of the HBP in hyperglycemia-mediated glycosylation of fibronectin in A549 cells. Furthermore, overexpression of GFAT significantly increased mRNA levels of IIICS domain of FN in cells cultured in NG medium and HG condition (Fig. 4D). The mRNA levels of GalNAc-T6 were slightly, but not significantly increased (Fig. 4E) indicating that the increased of onfFN in GFAT-overexpressing cells may occur through the elevated concentration of UDP-GalNAc and IIICS domain (acceptor substrate) and not because an increase of ppGalNAc-T6. Notably, overexpression of GFAT significantly increased the mesenchymal markers N-cadherin (Fig. 4F,G) and vimentin (Fig. 4F,H), indicating that influx of glucose through HBP triggers the EMT process in A549 cells.

## Discussion

Several works, over the past years, have demonstrated that elevated glucose levels induce increased expression of FN and TGF-β production by different cell lines [30]–[32]. TGF-β is a potent and known EMT inducer, and the emergence of mesenchymal markers, including FN is closely associated with EMT process. Recent studies bring to light the involvement of a key *O*-glycosylation in the IIICS domain of human FN forming the onfFN during the EMT process [22]. Importance of glycosylation in this process was reinforced by showing that knockdown of ppGalNAc-T6 inhibits onfFN biosynthesis and EMT in human prostate epithelial cells [22]. In addition, *O*-glycosylation of FN by ppGalNAc-T6 plays a function in the EMT progression in mammary epithelial cells [14]. Further work shows that purified onfFN, from human hepatoma HUH-7 cells overexpressing the *ppGalNAc-T6* gene, but not the non-glycosylated, so called “normal” FN (norFN), induces EMT in human lung cells A549 and NCI-H358 [33]. Here, we demonstrate, for the first time, that HG condition induces EMT-like events in A549 cells and increases O-glycosylation of FN (onfFN) with enhanced mRNA expression of FN splice forms containing the IIICS domain. Furthermore, we shown that HBP may have an important role on EMT process, since the overexpression of GFAT in A549 cells potentiated the expression of vimentin and N-cad, as well as the appearance of onfFN, which was determined by FDC-6 binding. Further studies need to be realized, but it is possible to speculate that: (i) HG condition induces endogenous TGF-β secretion and EMT events (morphology changes, emergence of mesenchymal markers and high cell motility) in A549 cells; (ii) endogenous TGF-β induces onfFN expression since the pre-treatment of the cells with anti-TGF-β was able to compromise the FDC-6 binding; (iii) HBP directly participates of onfFN biosynthesis; (iv) HBP modulates the EMT process in A549 cells as GFAT-overexpressing cells present high levels of the mesenchymal markers (vimentin and N-cad). Synergistic effect of the HBP with endogenous TGF-β and onfFN produced in HG condition culminate in the induction of EMT. Co-regulatory function of FN variants with TGFβ has also been reported [33], [34].

The basis for the role of onfFN during EMT is currently under investigation in our lab. One hypothesis is that FN glycosylation might modify signaling pathways involved in epithelial cell contact with ECM, leading to loss of tissue association influencing tumorigenesis [35]. Noteworthy is that changes in the expression levels of ppGalNAc-T family members and in the structures of *O*-linked glycans have been associated with a number of human diseases, including immunodeficiencies and cancer [36], [37]. In addition, these data raise the possibility that hyperglycemia may contribute to the increased risk of cancer in diabetes [38] by causing increased expression of onfFN. Studies have demonstrated that diabetic patients have poor prognosis when compared with non-diabetic cancer patients [39], what may be explained by aberrant glycosylation of cancer cells, which is related to metastatic malignance. Elucidation of the various signaling pathways mediated by hyperglycemia and excess hexosamines may allow the development of novel targets for therapeutic intervention and treatment of patients with idiopathic pulmonary fibrosis (IPF). IPF is a lethal lung disorder characterized by fibroblast accumulation, ECM remodelation, which leads to distortion of alveolar architecture, progressive decline in lung function, and untimely death [40]. The disease is likely the result of complex interactions between genetic and environmental factors. Evidence suggests that certain environmental factors, such as diabetes mellitus may increase the risk of developing IPF [41]. A previous report demonstrated that FN deposition in alveolar epithelial cell induces EMT *in vivo* during pulmonary fibrosis [42]. However, no distinction between total FN and onfFN was made.

How the HBP induces EMT process is not completely understood yet. One hypothesis is that HBP has its effects on EMT, at least in part, through TGF-β secretion. Studies in porcine mesangial cells show that high glucose causes a dose-dependent increase in the production of TGF-β through HBP [30]. Further work has provided evidence for the molecular mechanism linking high glucose-enhanced HBP activity with upregulated TGF-β promoter activity [43]. High glucose causes an accumulation of the upstream stimulatory factors (USF) in the nucleus of mesangial cells, leading to upregulation of TGF–β expression via enhanced binding of USF proteins to the TGF-β promoter. Another hypothesis is through protein O-GlcNAcylation. The substrate for this posttranslational modification of proteins is UDP-GlcNAc, the major product of the HBP. Growing evidence has linked aberrant O-GlcNAcylation to cancer [10], [44]. However, only one study shows that O-GlcNAc participates in the molecular mechanism involved in EMT [16]. O-GlcNAcylation at serine 112 of Snail, the repressor of E-cadherin, blocks its phosphorylation by GSK3β and protects Snail from ubiquitylation and degradation, Hyperglycaemic condition enhances O-GlcNAc modification and initiates EMT by transcriptional suppression of E-cadherin through Snail [16]

Together our data show, for the first time that high glucose induces EMT and production of onfFN. These data imply that metabolite availability to the HBP exerts control over gene expression and modulates cell surface glycosylation. Furthermore, our data suggest that changes in glucose uptake alter epithelial cell communication with neighboring cells and ECM, which results in loss of tissue organization and contributes to tumor formation and progression.
